# User Identification Using Gait Patterns on UbiFloorII

**DOI:** 10.3390/s110302611

**Published:** 2011-03-01

**Authors:** Jaeseok Yun

**Affiliations:** U-embedded Convergence Research Center, Korea Electronics Technology Institute, 68 Yatap-dong Bundang-gu, Seongnam, Korea; E-Mail: jaeseok@keti.re.kr; Tel.: +82-31-789-7581; Fax: +82-31-789-7589

**Keywords:** user identification, gait recognition, walking pattern, stepping pattern, UbiFloorII

## Abstract

This paper presents a system of identifying individuals by their gait patterns. We take into account various distinguishable features that can be extracted from a user’s gait and then divide them into two classes: walking pattern and stepping pattern. The conditions we assume are that our target environments are domestic areas, the number of users is smaller than 10, and all users ambulate with bare feet considering the everyday lifestyle of the Korean home. Under these conditions, we have developed a system that identifies individuals’ gait patterns using our biometric sensor, *UbiFloorII*. We have created UbiFloorII to collect walking samples and created software modules to extract the user’s gait pattern. To identify the users based on the gait patterns extracted from walking samples over UbiFloorII, we have deployed multilayer perceptron network, a feedforward artificial neural network model. The results show that both walking pattern and stepping pattern extracted from users’ gait over the UbiFloorII are distinguishable enough to identify the users and that fusing two classifiers at the matching score level improves the recognition accuracy. Therefore, our proposed system may provide unobtrusive and automatic user identification methods in ubiquitous computing environments, particularly in domestic areas.

## 1. Introduction

For the past decade, we have increasingly depended on computers to store and process information pertinent to our daily lives. Thus, an effective method for securing access control to computers has been very important. In conventional computer systems, surrogate representations of identity such as a password and personal identification numbers (PINs) have proven successful. However, because passwords and PINs can be easily guessed, observed, or forgotten, they are not very practical or secure. Moreover, ubiquitous computing environments necessitate reliable user identification through automatic, transparent, and often remote means.

One possible solution to providing automatic, secure, and user-friendly personal identification lies in the area of biometrics, which refers to the automatic recognition of people based on their distinctive anatomical (e.g., face, fingerprint, iris, retina, hand geometry) and behavioral (e.g., signature, gait) characteristics [1]. Of these characteristics, we focus on gait, which is a particular way or manner of moving on foot. Considerable evidence that gait is unique in its ability to determine one’s identity [2,3], thus supporting the use of one’s gait in recognition, has been reported in other domains such as biomechanics, mathematics, and psychology. Gait is attractive as a biometric identifier because it is unobtrusive and typifies the motion characteristics specific to an individual because it can be detected and measured at both a low resolution and a long distance [4]. In this study, we present the practical design of a floor system and a user identification method that extracts various distinguishable features from the gaits of various users over the floor system.

We assume that the target application is a domestic environment, the number of users is smaller than 10, and all users walk in bare feet, a typical Korean custom in the home. Under these assumptions, we have created a floor-based system, UbiFloorII, which consists of a large number of photo interrupter sensors in wooden tiles. We have gathered walking samples while the experimental participants walk over the floor and extracted various features from the walking samples. In UbiFloorII, we employed not only the walking pattern such as stride length but also richer information on the human gait such as cadence or stepping pattern. We collected walking samples from ten participants that maintained their ordinary walking style. These walking datasets provide the input for the training and testing procedure of multilayer perceptron as well as other classifiers for evaluation. We next developed feature extraction procedures using MATLAB, from which we could extract both the walking and stepping patterns from the walking samples. For each of the patterns, we trained the neural network with supervised learning, in which the learning rule was provided with a set of examples of proper network behavior, and then identified unknown walking samples with the well-trained neural network. We also implemented the neural network using MATLAB together with the feature extraction procedures. In order to show the stability of our identification method, we performed experiments with different classification methods including support vector machine, *k*-nearest neighbor algorithm, decision tree, decision table, and Bayes net. Finally, inspired by multi-modal biometric identification systems, we explored the fusion of the walking pattern and the stepping pattern at the matching score level for robust identification performance.

The proposed design of the floor-based identification system—given in terms of sensors, floor tiles, data acquisition and transmission, feature extraction module, and user identification module—appears to be appropriate for a laboratory environment but not a home environment. However, we discuss the remaining challenges for developing practical floor systems that can be adopted in a real home as a device that identifies its occupants. From these experiments, we conclude with confidence that a device that identifies individuals based on their walking and stepping patterns extracted from gait over the floor systems is feasible in practical terms.

The organization of the paper is as follows. Section 2 introduces various user identification systems using gait. Section 3 demonstrates a variety of features in human gait available for user identification. Section 4 describes the UbiFloorII systems. Section 5 explains the feature extraction procedures and user identification methods. Section 6 presents our data collection procedure and the feature sets we used. Section 7 presents experimental results with our proposed methods and the walking samples gathered with the floor system we developed. Section 8 presents the evaluation study for performance analysis and the fusion of walking pattern and stepping pattern. Section 9 discusses our observations on the results, and we offer concluding remarks in Section 10.

## 2. Related Work

Gafurov has demonstrated a good survey of biometric gait recognition based on the various approaches, including machine vision, floor sensor, and wearable sensor [5]. As summarized by Gafurov, depending on the type of biometric sensors, gait recognition systems are classified into three main categories: (i) vision-based approach, (ii) floor-based approach, and (iii) portable sensor-based approach.

### 2.1. Vision-Based Gait Recognition

Vision-based gait recognition focuses on recognizing an individual with various features extracted from a video-sequence of the person walking. In comparison with other biometric features such as fingerprints, vision-based gait recognition has the advantage of being unobtrusive. For example, iris recognition systems have an obtrusive interface because an individual must exhibit specific behavior (e.g., gazing in a specific direction or remaining still during a recognition latency time) to be identified. However, vision-based gait recognition systems require no individual contact other than walking. So far, the gait is probably the only perceivable biometric feature from a great distance and at a low resolution in comparison with face recognition systems. Nixon *et al.* presented an extensive survey of the various vision-based identification methods based on human gait [6]. Most current vision-based approaches are based on analysis of silhouettes in the sequences of images of walking human subjects. They may be explicitly classified into two main categories: (i) model-based analysis and (ii) model-free analysis.

The model-based analysis aims to explicitly model the human body or motion, and they usually perform model matching in each frame of a walking sequence so that the parameters such as trajectories are measured on the model. BenAbdelkader *et al.* estimated the cadence and stride length from low-resolution video based solely on the periodicity of the walking person and a calibrated camera [7]. Bobick and Johnson extracted relative body parameters from action of waking to describe the subject’s body and stride [8]. Tanawongsuwan *et al.* deployed joint angle trajectories extracted from markers placed on joint positions in the legs and on the thorax [9]. Yam *et al.* explored the intimate relationship between walking and running that was expressed as a mapping based on the idea of phase modulation [10]. Zhang *et al.* used the change in orientation of human limbs [11].

The model-free analysis establishes a correspondence between successive frames based on the prediction or estimation of features related to position, velocity, shape, texture, and color. Kale *et al.* considered the width of the outer contour of a binarized silhouette [2] while Sundaresan *et al.* considered the entire binary silhouette itself [12]. Vega and Sarkar used the change in the relational statistics among the detected image features, removing the need for object models, perfect segmentation, or part-level tracking [13]. Liu and Sarkar developed an average silhouette as the simplest recognition feature [14]. Collins *et al.* deployed key frame analysis for sequence matching with innate viewpoint dependence [15]. Lee and Grimson deployed ellipsoidal fits to human silhouettes [16]. The feature vector is composed of parameters of moment features in image regions derived from silhouettes. Bhanu *et al.* considered kinematic and stationary features by estimating 3D walking parameters by fitting a 2D kinematic model to 2D silhouettes [17]. Similarly, Han *et al.* used gait energy image formed by averaging silhouettes [18].

### 2.2. Floor-Based Gait Recognition

The main difference between the floor- and vision-based approaches lies in the biometric sensor used in the vision-based approach: a camera. This unobtrusive and transparent interface of vision-based gait recognition provides a strong advantage over other contact-type biometric recognition approaches such as iris recognition. Unfortunately, however, a camera is not always an optimal approach, as it is sensitive to environmental factors such as shadow and light intensity. Moreover, camera surveillance can compromise an individual’s privacy. Such an intrusion in privacy-sensitive environments such as one’s home has been the target of criticism in vision research. In the floor-based approach, by contrast, a floor is used as a biometric sensor that gathers the various features of one’s gait. Therefore, the floor-based approach not only preserves individual privacy but also withstands the effects of shadow and light. Like the vision-based approach, floor-based gait recognition begins from the notion that gait is observable, distinguishable, and idiosyncratic. Because a floor is the biometric sensor, measurable information is obtained from only the soles of an individual’s feet. Thus, the features for recognition are somewhat different from those of the vision-based approach.

Stride length and cadence are attributed to physical makeup such as height, body mass, and the lengths of limbs. Actually, many vision-based studies have examined how stride length and cadence in human walking can be employed for gait recognition [7,19,20]. Demonstrating the effectiveness of stride length and cadence in discriminating the gait of individuals, these studies contended that stride length and cadence could be used as biometric identifiers. They estimated cadence and stride length from a video-sequence by using the periodicity of the walking person and calibrated cameras. This style of human recognition provided the motivation for the research into the feasibility of a floor-based identification system in which walking features such as stride length and cadence could be employed. The first use of walking features as a biometric identifier in floor-based gait recognition systems was UbiFloorI developed in [21]. Strictly speaking, the feature they used was not stride length but center positions of footprints over multiple footsteps. However, considering the longitudinal and transversal differences between consecutive center positions of footsteps represent the variation of stride length and dynamic range respectively, UbiFloorI might be the first floor-based gait recognition system exploiting walking features including stride length and dynamic range. UbiFloorI consists of 144 inexpensive ON/OFF switch sensors (14 cm by 2.5 cm) fitted onto cushioned carpet and a data acquisition (DAQ) board for measuring and transmitting the data from the sensors. They extracted walking patterns, including stride length, dynamic range, and foot angle from a dataset of switch sensors and use a neural network to identify unknown walking samples. The experimental results showed about 90% recognition accuracy with 10 subjects. UbiFloorI provides users with a transparent and user-friendly interface because the only task users need to do is to walk naturally. However, time information such as stance and swing time (or cadence) was unavailable because of the unique mechanical characteristics of the switch sensors and the low resolution of the floor. Middleton *et al.* developed a prototype floor sensor (0.5 m by 3 m) composed of 1536 force sensitive resistors (FSR) and 3 PIC 16F84A microprocessors [22]. They collected dataset from 15 individuals and extracted walking pattern such as stride length and cadence and stepping pattern such as time on toe to time on heel ratio. Based on the features, they achieved an 80% recognition rate. More recently, Qian *et al.* developed a high-resolution pressure sensing floor to obtain 1D pressure profile and 2D position trajectories of the centers of pressure (COP) of both feet, forming a 3D COP trajectories over a footstep. Based on the 3D COP trajectories, they extracted various features such as stride length and mean pressure, and showed an average recognition rate of 92.3% with walking dataset collected from 11 subjects and Fisher linear discriminant classifier [23,24].

When a body is in contact with the ground, the downward force due to gravity is reflected back onto the body as a reaction. This reflected force is referred to as *ground reaction force* (GRF). Several attempts to use GRF for gait recognition can be seen in [25–27]. However, the focus of these efforts has been in diagnose and therapy rather than recognition and identification. The first attempt to deploy GRF for human recognition was the active floor [28], which consists of a 4 × 4 array of load cells. Each load cell supports the corners of four adjacent floor tiles. Addlesee extracted features from the vertical ground reaction force traces derived from an individual’s footsteps and used a hidden Markov model to recognize differences in their stepping patterns. Experimental results showed that the active floor correctly identified the footsteps of 15 subjects with 91% accuracy. Orr and Abowd’s work with the smart floor resembles that with the active floor [29]. They extracted 10 features from the vertical ground reaction force profiles of individuals and used the nearest neighbor search in 10-dimensional feature space to identify unknown footsteps. The method correctly identified the footsteps of the 15 subjects with 93% accuracy. Suutala *et al.* demonstrated a floor composed of simple binary switch sensors to detect footsteps and extract walking characteristics, and showed 84% total recognition rate with walking profiles collected from 9 subjects and a Bayesian approach using a Gaussian Process classifier [30].

Another method of identifying a person through gait recognition is using a static footprint, which is a footprint left after a person passes. Kennedy showed the possibility of identifying individuals through the pressure areas on the soles of their feet [31]. Nakajima measured the pressure distribution of a footprint with a pressure-sensing mat [32]. They showed an 85% recognition rate with 10 subjects. In this study, they assumed that the footprint-based identification and verification system would not work for the security application, but it could work for personal recognition in a small group such as that found in a home environment. Unlike the static footprint, a dynamic footprint can be defined differently depending on its biometric sensors. An example of a dynamic footprint is the GRF profile mentioned above. Jung defined a dynamic footprint as a COP (center of pressure) trajectory while stepping heel-strike to toe-off [33]. They measured a quantized COP trajectory from a mat-type pressure sensor and used a hidden Markov model to create probability models for each foot of a user. Their experimental results showed an 80% recognition rate with 8 subjects. In this paper, we present a new dynamic footprint, an array of transitional footprints over the floor we developed for user identification.

### 2.3. Portable Sensor-Based Gait Recognition

The most recent approach in gait recognition is the portable sensor-based approach, in which accelerometers are normally used as biometric sensors. Like the floor-based approach, the accelerometer-based approach has several advantages. One is that it has an unobtrusive interface, and it does not compromise a user’s privacy. On the other hand, it requires users to carry or attach an accelerometer and a motion-recording device on their bodies for identification. In the sensor-based method, accelerometers attached to several parts of the body such as the hip, the lower leg, or the waist, measure acceleration signal characteristics while the user is walking. Features are extracted from the output of the device accelerations in vertical, forward-backward, or sideways directions. Examples of accelerometer-based gait recognition systems include [34–36].

## 3. Features in Gait

**Figure 1.**
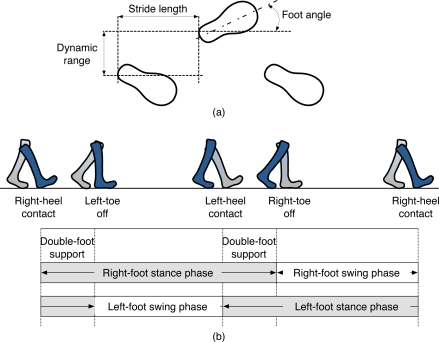
Examples of walking pattern in gait: **(a)** stride length, dynamic range, foot angle **(b)** stance time and swing time.

We may assume that the human gait motion consists of a sequence of footsteps that an individual follows while walking. Thus, we expect that some distinguishable characteristics with respect to the footsteps in the sequence can be extracted and used for recognition by considering the spatiotemporal variations of the observations. Through this paper, we define *walking pattern* as a spatiotemporal variation (e.g., stride length, dynamic range, foot angle, and stance and swing time that can be extracted from a sequence of an individual’s footsteps, illustrated in Figure 1.

**Figure 2.**
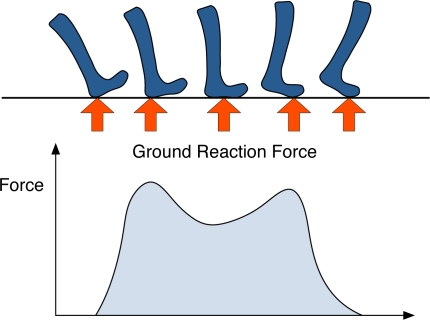
An example of stepping pattern in gait: ground reaction force.

*Stepping pattern* is defined as a temporal variation (e.g., transitional footprint and ground reaction force) that can be extracted from an individual’s footsteps during the stepping heel-strike to toe-off, illustrated in Figure 2. Thus, this paper will demonstrate whether the walking and stepping patterns extracted from one’s gait over our developed floor systems can be employed to identify the participants in domestic environments.

## 4. UbiFloorII System

**Figure 3.**
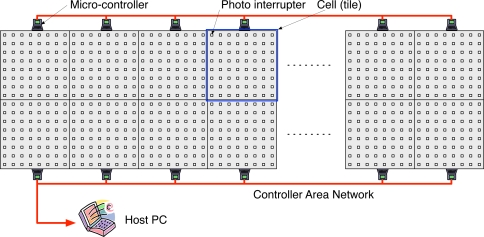
Overall structure of UbiFloorII.

To acquire a dataset of the footsteps of the experimental participants walking normally, we have developed the UbiFloorII system composed of photo interrupter sensors and wooden floor tiles. Figure 3 shows the overall structure of the UbiFloorII system, which consists of a 12 × 2 array of wooden tiles, each of which measures 30 cm × 30 cm and contains 64 uniformly-arranged photo interrupter sensors. A photo interrupter is non-contact and can convert the extent of reflective light to corresponding voltage. In our system, if an obstacle exists within detectable distance (3 mm), it generates 0 V voltage output, otherwise 5 V. We use DG-105 from Kodenshi Corp. and it measures 5 mm × 5 mm.

**Figure 4.**
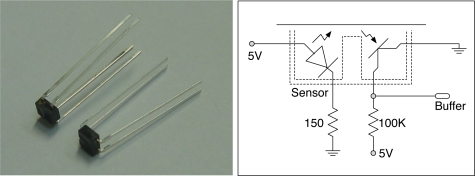
Reflective photo interrupters (**left**) and electric circuit (**right**).

**Figure 5.**
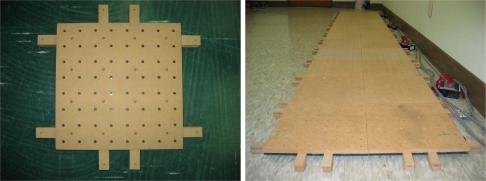
A wooden tile composed of 64 photo interrupters (**left**) and UbiFloorII (**right**).

Figure 4 shows the appearances of the photo interrupters and a corresponding electric circuit. A micro-controller is responsible for data acquisition from a corresponding tile and transmits the obtained information to the host PC through the CAN (controller area network) cable. Then, the host PC extracts features from the data and using a well-trained neural network, recognizes the user. With the transparent and user-friendly interface inherited from UbiFloorI, UbiFloorII can more easily be extended and maintained due to its modularized architecture. Figure 5 shows a wooden tile and the implemented UbiFloorII system. We have left out the full-detailed organization of the floor system, including the sensor, data acquisition, and data transmission [37].

## 5. User Identification with UbiFloorII

To identify individuals when they normally walk over the floor system, we need to extract the walking and stepping patterns, both distinguishable features mentioned previously. We presented the practical feasibility of the user identification method based on walking and stepping patterns over UbiFloorII in [37] and [38], respectively. Here, we present the full details of the walking and stepping features we used and the user identification method.

### 5.1. Walking Pattern Extraction

**Figure 6.**
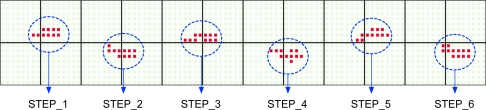
An example of searching footprints.

The software modules we develop for extracting a user’s walking pattern from data sets fall into two categories: (i) left footprint extraction and (ii) walking feature extraction. The left footprint extraction software is used to search for all footprints in the datasets received while one is walking over UbiFloorII, as shown in Figure 6.

We create an 8 × 4 footprint model that covers all probable footprints and choose three features as follows:

- The *X* index of the backmost sensor in a footprint
- The *Y* index of the backmost sensor in a footprint
- The footprint model of a footstep

**Figure 7.**
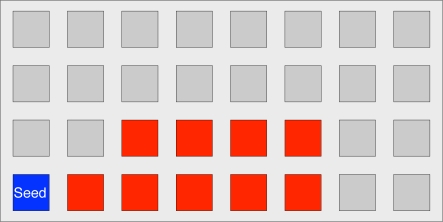
An example of a footprint model.

Figure 7 displays a footprint model extracted from the footprint in STEP 1 of Figure 6. As shown in Figure 7, the backmost sensor in a footprint becomes the seed sensor, from which the other features can be extracted.

We extract spatiotemporal walking features using the feature values obtained from left footprint extraction as inputs of the walking feature extracting software. We adopt seven walking features as follows:

- *FX* and *FY* : the physical *X* and *Y* coordinates of the backmost sensor in a footprint
- *com_FX* and *com_FY*: the compensated *X* and *Y* coordinates based on the footprint model
- *nSensor*: the number of pressed sensors in the footprint
- *fStart*: heel-strike time of the footstep
- *fEnd*: toe-off time of the footstep

**Figure 8.**
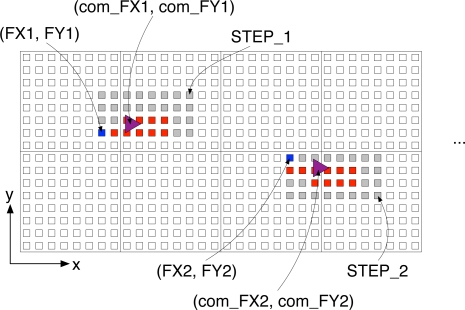
Walking feature extraction.

*FX* and *FY* represent the physical *X* and *Y* coordinates of the seed sensor in a footprint with the bottom-left corner of UbiFloorII as the origin. The coordinates *com_FX* and *com_FY* represent the center of the footprint based on the footprint model, as shown in Figure 8. Practically, *com_FX* and *com_FY* comprehend the user’s stride length, dynamic range, and foot angle. *fStart* and *fEnd* imply the user’s stance and swing time in walking. Finally, to create input vectors to the neural network, we need to generate the sequences of each walking feature in terms of the footsteps, such as [*com_FX*1, *com_FX*2, *com_FX*3, . . . ].

### 5.2. Stepping Pattern Extraction

**Figure 9.**
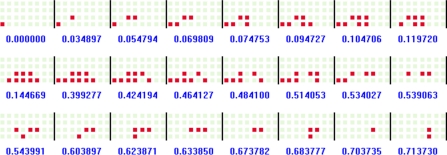
An array of transitional footprints.

**Figure 10.**
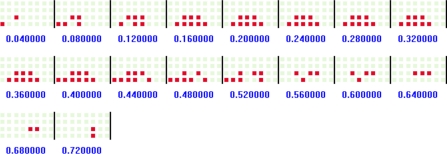
An array of sampled transitional footprints.

To extract the stepping pattern in each footprint, we need to analyze the temporal variation of each footstep. Transitional footprint extracting software is used to analyze the variation of transitional footprints from heel-strike to toe-off in each footstep. Based on the left footprint obtained from the walking pattern extraction procedure, we obtain an array of transitional footprints in terms of the occurrence times of the events (i.e., changes in the states of the sensors). The array of transitional footprints extracted from the STEP 1 footprint in Figure 6 is shown in Figure 9, where the digit values denote the occurrence times (seconds) of the events. Finally, to make a standard of the stepping pattern, we extract the array of sampled transitional footprints at a uniform sampling time from the array of original transitional footprints. Figure 10 shows the array of sampled transitional footprints extracted from Figure 9 with a sampling time of 0.04 (s).

For user identification, we adopted two stepping features as follows:

- The left footprint
- The array of sampled transitional footprints

To create input vectors to our neural network, we need to convert the left footprint and the array of sampled transitional footprints to the vectors. In the case of the left footprint, each gray square in Figure 7 is represented by a “−1”, and each dark square is represented by a “1”. Then, to create the input vectors, we scan the 8 × 4 left footprint of one column (*i.e.*, four sensors) at a time. For example, in Figure 7, the input vector to the neural network looks like [−1 −1 −1 1 −1 −1 −1 1 . . . ]. In the case of the sampled transitional footprints, the process is similar to that in the case of the left footprint, except that the number of rows is 8 multiplied by the number of elements in the array (e.g., 18 in Figure 10).

### 5.3. User Identification

We use multilayer perceptron networks to identify individuals based on the extracted walking and stepping features [39]. Inspired by biological neurons, neural networks (along with their components) consist of simple neurons, and the connections among them determine the function of the network. The network is trained to perform special tasks such as modifying the weights and biases of a network by applying the learning rule. The learning rule used in this work is the back-propagation algorithm. If well-trained with the back-propagation learning algorithms, the multilayer perceptron can correctly classify the samples even if they are unknown during network training.

**Figure 11.**
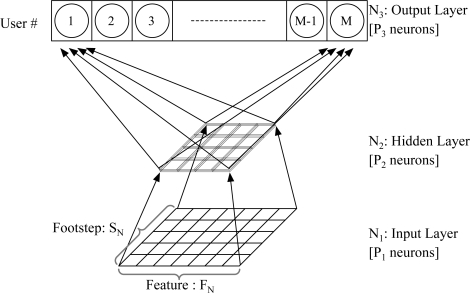
Structure of the neural network for user identification.

The neural network that we deploy for identifying users by their walking patterns has the same structure as the neural network for identifying users by their stepping patterns, and both neural networks consist of three layers, as shown in Figure 11: (i) an input layer *N*_1_ with *P*_1_ neurons, (ii) a hidden layer *N*_2_ with *P*_2_ neurons, and (iii) an output layer *N*_3_ with *P*_3_ neurons. In the neural network for identifying an individual’s walking pattern, the number of neurons in the input layer is equal to the number of walking features *F_N_* times the number of walking steps *S_N_*. Likewise, in the neural network for identifying an individual’s stepping pattern, if the number of the elements in the step features is *F_N_* and the number of footsteps used is *S_N_*, then the total number of input neurons, *P*_1_ is equal to *F_N_ × S_N_*. The number of neurons *P*_2_ in the hidden layer *N*_2_ is selected by experiments and the number of neurons *P*_3_ in the output layer *N*_3_ is equal to the number of users *M*. Because the neural network is trained with supervised learning, the learning rule is provided with a set of examples (*i.e.*, the training set) of proper network behavior. We have used sigmoid activation functions trained with backpropagation.

The training procedure for our network is as follows. In the neural network for walking pattern-based identification, we need to perform preprocessing; that is, we must normalize the input values to the network. In contrast, in the neural network for stepping pattern-based identification, the preprocessing procedure is not needed in our neural network because all elements of the input vectors are digital values. Thus, the input vectors converted from the left footprints or the arrays of sampled transitional footprints will be direct inputs to the neurons of the input layer in our network. The learning rule is used to adjust the weights and biases of the network in order to move the network outputs closer to the targets. Given a test sample for the well-trained network, we can choose the index number of the output node with maximum output values as the user’s identification number.

(1) $$\mathrm{User}\_\mathrm{Number}=\max\left(O_{1},O_{2},\ldots ,O_{M}\right)$$

where *O_i_* denotes the output values of the *i*-th node, and *M* is the number of users (*i.e.*, 10).

## 6. Experiment Setup

### 6.1. Data Collection and Experimental Conditions

Our experiments consisted of capturing walking samples from 10 experimental participants volunteered from the academic community. Data was collected from 10 males. Participants ranged in age from 27 to 35 (see Table 1). For each subject, we gathered 50 walking samples. We collected a total of 500 walking samples from each participant walking on the UbiFloorII system. Depending on the user’s stride length, it takes from five or six footsteps to cross UbiFloorII. Therefore, we consider only the first five steps (STEP_1 – STEP_5). All the subjects were asked to walk as normally as possible and given the option to listen to soothing music. Moreover, they were asked to walk with bare feet, reflecting the daily custom followed in the Korean homes. In order to mitigate any variation in their gaits, we imposed all of these conditions on the participants while collecting the walking samples.

### 6.2. Walking Feature Sets

To verify the dominant walking features, we use five feature sets as inputs to the network. Table 2 shows the combinations of these features. In Case 1, coordinates *FX* and *FY* are the inputs to the network. This case is used as the standard for evaluating the results with those of the other feature sets. The other feature sets comprise the combinations of our walking features.

### 6.3. Stepping Feature Sets

To verify the dominant footsteps in stepping features, we use four feature sets as inputs to the network. Table 3 shows the combinations of the features. In Cases 1 and 2, the odd and even footsteps of the first four footsteps, respectively, are the inputs to the network. In Case 3, the first two footsteps are the inputs to the network, and in the last case, all four footsteps are inputs. In the experiments using the array of sampled transitional footprints, various input arrays can be extracted from the left footprint in terms of the sampling times. Thus, we use five sampling times, as shown in Table 4, in which the elapsed times of all footsteps is assumed to be less than 0.8 s.

## 7. Experiment Results

All the experiments based on the neural network in this study are carried out using the Neural Network Toolbox developed by the MathWorks, Inc.

### 7.1. User Identification Based on Walking Pattern

**Figure 12.**
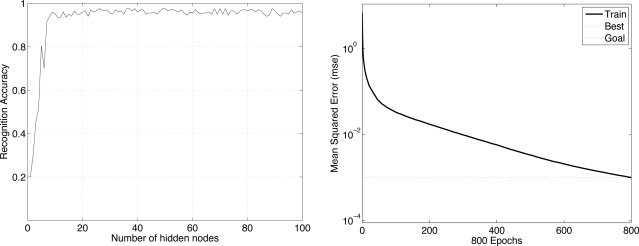
Results of deciding the number of hidden nodes (**left**) and the epoch and goal (**right**) for walking pattern-based identification.

We first demonstrate how the number of hidden nodes influences the performance of the neural network. In an effort to decide the optimal number of hidden nodes, we run an experiment in which we increase the number of hidden nodes while keeping other parameters fixed and observe the resulting recognition accuracy. The results of one experiment with *com_FX*, *com_FY*, *fStart*, and *fEnd* features are shown in Figure 12. The left side of Figure 12 shows that about 30 hidden nodes are sufficient to obtain about a 95% recognition rate. We also conduct experiments to decide the *epoch* and the *goal*. The right side of Figure 12 shows that after 800 epochs, the mean square error is smaller than 10^−3^, so this value is set to the goal.

We present the results of the test with our feature sets and a comparative analysis in Table 5. In this experiment, the recognition accuracies are obtained by averaging 10 simulation results changing the seed value that determines the initial values of weights and biases of the network. First, we can note that the compensation procedure for *com_FX* and *com_FY* results in about a 10% improvement in recognition accuracy. Considering Cases 2 and 3, Case 3 is worse than Case 2 (*i.e.*, without *nSensor*) because *nSensor* information has already influenced the compensation procedure for *com_FX* and *com_FY*. We are able to achieve about a 96% recognition accuracy when *com_FX*, *com_FY*, *fStart*, and *fEnd* features are used. Therefore, we conclude that the stance and swing time is also a dominant feature for user identification.

In [21], they chose the physical *X* and *Y* coordinates of the center of the footprint, which produced the stride length and the dynamic range for a given individual, as the walking pattern, illustrated in Figure 1(a). Even though they could not use stance and swing time information due to the low density of UbiFloorI, the experimental results exhibited a recognition accuracy of about 90%. For UbiFloorII, we used not only the features that we employed for UbiFloorI but also stance and swing time information, illustrated in Figure 1(b). To observe the potential of the new time information for improving recognition accuracy, we first performed the experiments without stance and swing time, which showed a recognition accuracy of approximately 90%, which is almost the same as that of UbiFloorI. However, we observed that the recognition accuracy could be improved to as much as 96% with stance and swing time. Here, we find it very important to note that except for stance and swing time, recognition accuracy with the previous walking pattern (the physical *X* and *Y* coordinates of the center of the footprint) might not improve any more with regard to the resolution of the floor.

### 7.2. User Identification Based on Stepping Pattern

**Figure 13.**
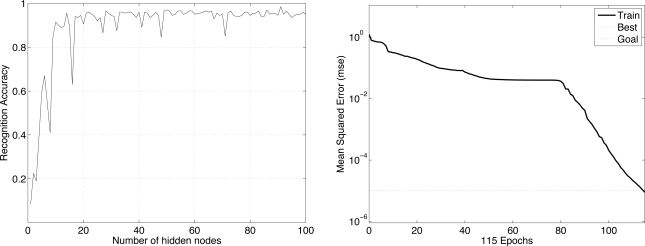
Results of deciding the number of hidden nodes (**left**) and the epoch and goal (**right**) for stepping pattern-based identification.

As we did when identifying users by their walking patterns, we first demonstrate how the number of hidden nodes influences the performance of the neural network. In general, the greater the number of hidden nodes, the longer the computation time. The results in this experiment with the arrays of sampled transitional footprints (Case 4, sampling time = 0.04 s) are shown in Figure 13, the left side of which shows that about 30 hidden nodes are sufficient for about a 90% recognition rate. We also conducted experiments to decide the epoch and the goal. The right side of Figure 13 shows that after 115 epochs, the mean square error (MSE) is smaller than 10^−5^, so this value is set to the goal. In all these experiments, we train the network using Powell-Beale and scaled conjugate gradient algorithms.

We present the results of the tests with our feature sets and a comparative analysis in Table 6. The first column shows the recognition accuracies with the left footprints, and the other columns show the recognition accuracies with the arrays of sampled transitional footprints in terms of various sampling times. In these experiments, the recognition accuracies are obtained by averaging the 10 simulation results changing the seed value. The experimental results with the left footprints show that the left footprint, which expresses the static shape of the sole of the user’s foot in UbiFloorII, is not distinctive enough to recognize individuals. In the experimental results with the arrays of sampled transitional footprints, most of them show a more than 80% recognition rate. We are able to achieve about 92% recognition accuracy with the arrays of the sampled transitional footprints (Case 4, sampling time = 0.04 s). Considering Cases 1, 2, and 3, two footsteps are not sufficient to achieve more than 90% recognition accuracy without regard to the order of the footsteps and sampling time. In addition, we can note that by shortening the sampling time for extracting the array of sampled transitional footprints from the array of original transitional footprints, the performance of recognition could be improved. Nevertheless, the small sampling time requires numerous inputs to the network, causing heavy computational load. Thus, we should consider limiting the sampling time for extracting the array of sampled transitional footprints.

In the UbiFloorII system, we chose static footprints and an array of sampled transitional footprints, or dynamic footprints, which represent the temporal variation of the shape of the bottom of the foot making contact with the floor from heel-strike to toe-off in each footstep, as the stepping pattern, illustrated in Figure 2. The experimental results from the static footprints, which express the static shape of the sole of the user’s foot in UbiFloorII, showed that they are not distinctive enough for human recognition, an expected finding based on the previous study [32]. The experimental results with the array of sampled transitional footprints exhibited a recognition accuracy of about 92%, but some variation obviously occurred in the recognition accuracy as a result of the sampling time. By shortening the sampling time taken to extract, we determined that the array of sampled transitional footprints and the performance of recognition could be improved. Nevertheless, a small sampling time requires numerous inputs to the network, causing heavy computational load. Thus, we would have to consider limiting the sampling time for extracting the array of sampled transitional footprints.

The problem mentioned previously is the numerous input nodes in the neural network, causing heavy computational load, for example as in the case of the best recognition result,

(2) $$\begin{array}{lll} \#\;\;\text{of input nodes} & = & 32\;(\text{footprint model})\\ & * & \frac{0.8\;(\text{maximum stepping time})}{0.04\;(\text{sampling time})}\\ & * & 4\;(\#\;\;\text{of used footsteps})\\ & = & 2560 \end{array}$$

One solution to this problem is training in principal components analysis (PCA). PCA is a way of identifying patterns in data and expressing the data in such a way as to highlight their similarity and differences [40]. The purpose of PCA training is to obtain several principal components that represent the original stepping features from a high-dimensional measurement space to a low-dimensional eigenspace. PCA training has been employed on the vision-based gait recognition approaches [41–43]. In the floor-based gait recognition approaches, Suutala and Röning built a frequency domain presentation of GRF signal (camel-back curve) and deployed PCA to reduce high dimensionality of the amplitude spectrum presentations [44]. We adopted an analogous method to that presented in [42], reducing the original 2560-dimensional space to a vector with 248 coefficients. The accuracy of recognition with this PCA training technique was about 89%, which is reasonable considering the greatly reduced computational loads.

## 8. Evaluation

Although we have shown that the neural network classifier (multilayer perceptron) is working well on the stepping and walking pattern extracted from our dataset, we should perform experiments with different classification methods to be able to see the stability of the user identification systems. Among various available machine learning algorithms, We chose five classification methods in addition to multilayer perceptron we had used: instance-based learning (*k*-nearest neighbor algorithm), decision tree (C4.5), Bayes net, decision table, support vector machine. Generally, discriminative machine learning algorithms such as multilayer perceptron have shown better performance than generative models such as hidden Markov model (HMM) in footstep identification. Support vector machine is chosen as one of the state-of-the-art discriminative method with a good performance in many applications. We chose the simple *k*-nearest neighbor algorithm from instance-based learning algorithms, and decision tree and decision table from rule-based learning algorithms. In addition, Bayes net is chosen as one of generative models to show its performance in gait recognition experiments.

All the experiments based on these classifiers in the evaluation study were carried out using Weka developed by Machine Learning Group at University of Waikato [45]. Weka is a data mining tool with open source machine learning software in Java. It supports all the classification methods we chose for evaluation, including multilayer perceptron we used with MATLAB in the previous section. Therefore, we would be able to compare the experimental results among various classifiers as well as between machine learning tools, *i.e.*, MATLAB and Weka. We also used Eclipse to build and run a Java program based on the Java classes supported by Weka [46].

### 8.1. Walking Pattern

Table 7 summarizes mean and standard deviation for classification accuracy over the selected classification methods based on walking pattern we extracted from the walking samples. We used 10 times 10-fold cross-validation, *i.e.*, 10 different 10-fold cross-validation experiments with the same learning method and dataset, averaging the 100 experimental results. Overall, recognition accuracy is highest for multilayer perceptron, which is consistent with the experimental result with MATLAB in the previous section. Support vector machine is the second most accurate algorithm, and *k*-nearest neighbor method shows good performance as well. However, rule-based learning algorithms, particularly decision table shows lower accuracy compared to other algorithms. After looking into the confusion matrix, we know that this is because some portion of the walking samples of each subject was misclassified as a particular user (User_0_), *i.e.*, False Positive rate of the subject is considerably higher than others.

Table 8 shows an aggregate confusion matrix for multilayer perceptron classifier based on 10 subjects’ walking pattern. Recognition accuracy for User_0_ is lower than others because some portion of the walking samples of User_0_ was misclassified as User_1_, User_3_, User_4_, User_9_. We can also know that User_2_ and User_3_ are confused with User_7_ and User_6_ respectively since the subjects may have walked in similar style while collecting walking samples. However, it should be noted that the confusion between the users might not have been occurred by their height since there is very little correlation between them as shown in Table 1.

### 8.2. Stepping Pattern

Table 9 summarizes mean and standard deviation for classification accuracy over the selected classification methods based on stepping pattern we extracted from the walking samples. We used 10 times 10-fold cross-validation, *i.e.*, 10 different 10-fold cross-validation experiments with the same learning method and dataset, averaging the 100 experimental results. Overall, recognition accuracy is highest for support vector machine. It should be noted that this result is not very surprising because the past work in [44] recognized 11 subjects’ different single-footstep feature presentation with support vector machine classifier, and its recognition accuracy was higher than multilayer perceptron neural network. Multilayer perceptron is the second most accurate algorithm, and its accuracy is consistent with the experimental result with MATLAB in the previous section. As in case of walking pattern-based user identification, rule-based learning algorithms show lower performance than others because False Positive rate of User_0_ is considerably higher than others. It also should be noted that Bayes net shows weak performance compared to the discriminative model, which is consistent with past works on footstep-based recognition.

Table 10 shows an aggregate confusion matrix for support vector machine based on 10 subjects’ stepping pattern. We can know that User_5_ is often misclassified as User_4_ since the subjects may have foot-stepped in similar style while collecting walking samples. The confusion between users might not have been occurred by their foot size because there is very little correlation between them as shown in Table 1.

### 8.3. Data Fusion

In the above section, we showed that both the walking and stepping patterns extracted from the human gait can be employed for the biometric identification of an individual. However, the recognition accuracy could be improved by combining two classifiers. In the last decade, a substantial amount of research has been devoted to multimodal biometric systems [47,48]. Such systems exhibit high performance when the biometric features stem from totally disparate sources such as faces, voices, and fingerprints. In the floor-based gait recognition method, Suutala and Röning presented a way of improving classification accuracy and the adaptability based on the conditional posterior probability outputs of classifiers, *i.e.*, efforts to combine classifiers trained with different feature sets and to combine multiple footstep instances of a single person walking on the floor [44].

**Figure 14.**
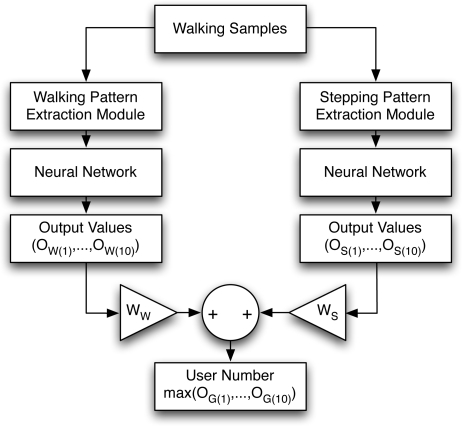
A flow chart of fusion at the matching score level for gait recognition.

Disparate biometric identifiers are fused using three basic techniques [49]: (i) fusion at the feature extraction level, (ii) fusion at the matching score level, and (iii) fusion at the decision level. The dimension of the feature vector of the stepping pattern is significantly higher than that of the walking pattern. Thus, it is not reasonable to concatenate the two feature vectors into a single vector for fusing at the feature level. Instead, we have adopted fusion at the matching score level. Figure 14 presents a flow chart of two classifiers combined by fusion at the matching score level for gait recognition.

In our case, each neural network generates *±*1 max-min score values, so normalization of the output score is not necessary. We can combine the output score values from each system into a new score value using Equation (3):

(3) $$O_{G(i)}=W_{W}\times O_{W(i)}+W_{S}\times O_{S(i)}$$

where *O*_*G*(*i*)_ denotes new output values of the *i*-th node, and *W_W_* and *W_S_* represent the weighting parameters for the walking and stepping patterns, respectively. The weighting values *W_W_* and *W_S_* were chosen so that the recognition accuracy could be maximized by combining the two classifiers (in our experiment *W_W_* = 0.96, *W_S_* = 0.92). *O*_*W*(*i*)_ and *O*_*S*(*i*)_ depict the output values from the neural networks of the walking and stepping patterns, respectively. Finally, we can choose the index number of the output node with the maximum output values as the user’s identification number (See Equation (1)). With this fusion technique, we could achieve a recognition accuracy of about 99%. From the experimental results, however, we cannot claim that our gait recognition technique will almost perfectly recognize all the participants in a domestic environment. Instead, we choose to focus on the potential of combining two classifiers for the robust performance of the identification system since the human walk, or gait, is likely to vary over time.

## 9. Discussion

We compare the proposed method to the existing floor-based user identification systems. We have tabulated and summarized the design and performance of all the methods in terms of sensors, features, the number of subjects, classifiers, and recognition accuracy in Table 11.

The sensors used in the floor-based gait recognition systems can be mainly classified into two classes: switch sensors (binary switch [21,44], photo interrupter [37,38]) and pressure sensors (load cell [28,29,32], ElectroMechanical Film [44,50–53], Force Sensing Resistor [22–24]). In early works, load cells were used to measure GRF profile over a footstep. Recently, fine-grained sensor technologies have been widely adopted in the gait recognition systems to capture pressure profiles over a footstep. However, it should be noted that the recognition accuracy of the user identification system depends mainly on the features extracted from raw dataset–of course, they are closely related to which sensors are used–and the classification methods rather than the sensor itself.

The features used in the floor-based gait recognition systems can be mainly classified into three classes: pressure profiles, 2D positional COP trajectories, and walking features such as stride length, cadence, and dynamic range. Among these features, the methods only using 2D positional COP trajectories showed weak performance [33]. However, when combining them with other features such as pressure profiles or walking features, the recognition accuracy was definitely improved as shown in [23,24]. Our transitional footprint is somewhat different from the dynamic footprint presented in [24,29,33,44]. While the dynamic footprints are a profile of the ground reaction force or the COP trajectory during footstepping heel-strike to toe-off, our dynamic footprint is composed of a sequence of transitional footprints while a user is walking. The grey-level image used in [30] is somewhat similar to our transitional footprints. While they extracted more elaborate features such as minimum, maximum, mean, standard deviation, and sum of components from the grey-level image, we provided the sampled *in situ* footprint images as the input features to the classifiers. As mentioned above, the characteristics of the dynamic footprint strongly depend on the sensor deployed in each floor system. Thus, if we consider that all user identification methods based on the various dynamic footprints showed reasonable performance, we can conclude that numerous other sensors could be deployed in floor systems to sense and extract much more distinguishable dynamic footprints for identifying individuals in domestic environments.

As we used 5 consecutive footsteps for gait recognition, multiple footsteps have been used to improve the recognition accuracy. In particular, it has been shown that the method in [53] deployed only footprint-based features over 5 consecutive footsteps (without walking features like stride length), achieving more than 95% recognition accuracy. They classified each footstep using a single classifier, combining the result of each classifier with sum and product rules. Instead, we concatenated the footprint images over multiple footsteps into a single feature vector, and then classified it with a single classifier. Although there would still be a question of which method is better in terms of recognition accuracy, the method of combining classifiers over multiple footsteps might be more practical in real environments because it would be able to start immediately identifying them once unknown footsteps are detected on the floor.

Various machine learning algorithms including hidden Markov model (HMM) [28,33,50,54], learning vector quantization (LVQ) [51] and distinction-sensitive LVQ (DSLVQ) [52], *k*-nearest neighbors (KNN) [29], multilayer perceptron (MLP) [21,37,38,44,53], Gaussian process (GP) [30], Fisher linear discriminant (FLD) [23,24], and support vector machine (SVM) [44] have been studied in the gait-based user identification systems. Generally, discriminative machine learning algorithms such as support vector machine have shown better performance than generative models such as hidden Markov model in footstep identification. In [44], Suutala and Röning presented the comparison for the recognition performance of the various classification methods including KNN, LVQ, RBF, MLP, and SVM, demonstrating MLP and SVM are the recommended classifiers in footstep-based user identification, being consistent with our experimental results. However, recently, Qian *et al.* used FLD, one of generative models, as the classifier, and they showed good performance of the method based on comprehensive features over multiple footsteps including pressure profiles, 3D COP trajectories, and stride length [24].

Unfortunately, previous research has shown that as gait databases continue to expand in size, it is conceivable that identifying an individual only by gait may become difficult. Thus, we assume conditions in which the target application is a home environment. However, we located several studies in which gait recognition was deployed for surveillance and for security in public areas. In these applications, gait provided only one of the multimodal biometric identifiers for the system, so it could serve as a useful filtering tool allowing one to narrow the search to a considerably smaller set of potential candidates. In contrast, in this study, we have focused on the identification techniques for unobtrusive interfaces and privacy-preserving devices rather than high-security systems. Thus, we have begun to recognize the feasibility of using our floor-based identification technique in home environments, accounting for reasonable accuracy, unobtrusive interface, and privacy-comfortable systems.

Although the floor systems we have developed presented the potential of serving as a sensor device for a gait-based identification system, we have identified several shortcomings that will limit its use to laboratory environments and preclude its use in home environments. For example, since UbiFloorII has a large number of holes drilled in its wooden tiles to house the photo interrupter sensors, so spilling water on the floor would obviously damage the electronic circuitry embedded inside UbiFloorII. Moreover, although UbiFloorII contains an electronic circuitry and micro-processor program that minimizes power consumption, it still continuously consumes considerable energy due to photo interrupter sensors. If an entire room is equipped with the floor tiles, it may be able to track multiple individuals. Nevertheless, considerable study will be needed to solve the problems caused from the overlapping of dynamic objects (e.g., activities of passing, gathering, or separating among occupants) and of dynamic and static objects (e.g., sitting on a chair). Future work that involves developing a new type of gait-based human recognition systems capable of tracking and identifying multiple occupants simultaneously should represent a significant, intriguing research area.

## 10. Conclusions

We have presented a method of identifying individuals by their gait patterns. We classified available features of gait into two categories: walking pattern and stepping pattern. The former is defined as the spatiotemporal variation in a sequence of footsteps such as stride length, dynamic range, foot angle, and stance and swing time; and the latter is defined as the temporal variation over a single footstep such as a dynamic footprint. We assume that the target application is a home environment, that the number of users is smaller than 10, and that all users walk with bare feet, reflecting the Korean custom. With these assumptions, we have developed a floor-based identification system using walking and stepping patterns that can be extracted from users’ walk over our biometric sensor, UbiFloorII. To collect walking samples, we have created UbiFloorII using photo interrupter sensors. We have also developed feature extraction modules and a user identification module using a neural network technique. Our findings show that the walking and stepping patterns extracted from the users’ gaits over the UbiFloorII are distinguishable enough to identify the users. We also found that by fusing two classifiers at the matching score level, the performance of the identification system may be robust against the likely variation in the human gait. With the advent of new low-power, fine-grained sensor technologies for measuring elaborate patterns of human gait or the discovery of a new distinguishable feature available for user identification, we expect the creation of a more accurate and robust gait-based human identification system in the near future.

## Figures and Tables

**Table 1.** Heights and foot sizes of the subjects.

**Subject**	**Height (cm)**	**Foot size (mm)**

1	168	255
2	173	260
3	165	260
4	172	275
5	168	265
6	180	280
7	173	255
8	175	260
9	180	265
10	168	260

**Table 2.** Classifications of walking feature sets.

**Case**	**Features sets**	**The number of inputs**

1	*FX*, *FY*	10
2	*com_FX*, *com_FY*	10
3	*com_FX*, *com_FY*, *nSensor*	15
4	*com_FX*, *com_FY*, *fStart*, *fEnd*	20
5	*com_FX*, *com_FY*, *fStart*, *fEnd*, *nSensor*	25

**Table 3.** Stepping feature sets in terms of used footsteps.

**Case**	**Used footsteps**	**The number of steps**

1	STEP_1, STEP_3	2
2	STEP_2, STEP_4	2
3	STEP_1, STEP_2	2
4	STEP_1, STEP_2, STEP_3, STEP_4	4

**Table 4.** Various sampling times for transitional footprints.

**Sampling time (s)**	**The number of elements of the array**

0.04	20
0.05	16
0.06	13
0.08	10
0.10	8

**Table 5.** Comparison of recognition accuracy of user identification by walking pattern.

**Case**	**Features sets**	**Recognition accuracy (%)**

1	*FX, FY*	80.75
2	*com_FX, com_FY*	89.05
3	*com_FX, com_FY, nSensor*	86.85
4	*com_FX, com_FY, fStart, fEnd*	96.20
5	*com_FX, com_FY, fStart, fEnd, nSensor*	95.20

**Table 6.** Comparison of recognition accuracy (%) of user identification by stepping pattern.

		**Sampling time (s)**				

	LEFT	0.04	0.05	0.06	0.08	0.10
**Case 1**	58.6	89.0	89.2	84.4	82.7	84.5
**Case 2**	60.7	89.3	82.4	80.7	73.4	80.3
**Case 3**	60.0	83.9	84.9	84.8	82.5	79.0
**Case 4**	68.1	92.0	91.9	91.3	88.5	86.0

**Table 7.** Summary of classifier results (mean *±* standard deviation). Comparison of recognition accuracy (%) of user identification by walking pattern.

**Classifier**	**Recognition accuracy (mean ± std)**

Multilayer Perceptron	96.64 ± 0.38
Instance-based Learning	94.08 ± 0.47
Decision Tree	88.30 ± 0.83
Bayes Net	90.86 ± 1.28
Decision Table	73.92 ± 1.36
Support Vector Machine	95.88 ± 0.33

**Table 8.** Aggregate confusion matrix for multilayer perceptron based on 10 times 10-fold cross-validation for 10 subjects’ walking pattern.

**User**_0_	**User**_1_	**User**_2_	**User**_3_	**User**_4_	**User**_5_	**User**_6_	**User**_7_	**User**_8_	**User**_9_	**← Classified as**
445	10	0	23	12	0	0	0	0	10	User_0_
5	478	0	4	10	0	2	0	1	0	User_1_
0	0	485	0	0	0	0	15	0	0	User_2_
22	0	0	463	1	0	11	0	0	3	User_3_
0	0	0	0	500	0	0	0	0	0	User_4_
0	0	0	0	0	500	0	0	0	0	User_5_
0	0	0	12	0	0	488	0	0	0	User_6_
0	0	11	0	0	0	0	489	0	0	User_7_
0	0	0	0	1	0	0	0	499	0	User_8_
10	0	0	5	0	0	0	0	0	485	User_9_

**Table 9.** Summary of classifier results (mean ± standard deviation). Comparison of recognition accuracy (%) of user identification by stepping pattern.

**Classifier**	**Recognition accuracy (mean ± std)**

Multilayer Perceptron	92.44 ± 0.28
Instance-based Learning	84.32 ± 0.48
Decision Tree	79.16 ± 1.55
Bayes Net	91.62 ± 0.37
Decision Table	57.38 ± 1.61
Support Vector Machine	95.61 ± 0.26

**Table 10.** Aggregate confusion matrix for support vector machine based on 10 times 10-fold cross-validation for 10 subjects’ stepping pattern.

**User**_0_	**User**_1_	**User**_2_	**User**_3_	**User**_4_	**User**_5_	**User**_6_	**User**_7_	**User**_8_	**User**_9_	**← Classified as**
497	0	3	0	0	0	0	0	0	0	User_0_
0	486	0	14	0	0	0	0	0	0	User_1_
11	0	487	0	0	0	0	10	0	0	User_2_
0	12	0	454	18	16	0	0	0	0	User_3_
0	0	0	10	466	24	0	0	0	0	User_4_
0	0	0	0	52	448	0	0	0	0	User_5_
0	0	0	0	4	0	496	0	0	0	User_6_
0	0	15	0	0	0	0	485	0	0	User_7_
0	11	17	0	0	1	0	0	471	0	User_8_
0	0	0	2	0	0	0	0	0	498	User_9_

**Table 11.** Performance comparison of all floor-based systems.

**Methods**	**Sensors**	**Features**	**Classifiers**	**Subjects**	**Accuracy**
Kennedy, 1996, [31]	Inked barefoot prints	Pressure areas on the soles of the feet	Physical matching	N/A	N/A
Addlesee, 1997, [28]	Load cells, floor	GRF discrete signal over a footstep	HMM	15	91.3
Orr, 2000, [29]	Load cells, floor	GRF profile features over a footstep	KNN	15	93.0
Nakajima, 2000, [32]	Load cells, mat	Direction and position of the footprints	Distance function	10	85.0
Yun, 2003, [21]	Switch sensors, mat	Foot centers over 5 consecutive footsteps	MLP	10	92.8
Jung, 2003, [33]	Pressure sensor, mat	2D COP trajectories over 2 consecutive footsteps, combine classifiers	HMM	8	64.0
Pirttikangas, 2003, [50]	ElectroMechanical Film, floor	Prototype vector via codebook for profile features	HMM	3	76.8
Pirttikangas, 2003, [51]	ElectroMechanical Film, floor	Prototype vector via codebook for profile features	LVQ	11	78.0
Jung, 2004, [54]	Pressure sensor, mat	2D COP trajectories over 2 consecutive footsteps, combine classifiers	HMM, NN	11	79.6
Suutala, 2004, [52]	ElectroMechanical Film, floor	Features from spatial, frequency domain over a footstep	DSLVQ	11	70.2
Middleton, 2005, [22]	Force Sensing Resistor, mat	Stride length, cadence, heel-to-toe ratio over 4 consecutive footsteps	N/A	15	80.0
Yun, 2005, [38]	Photo interrupters, floor	Foot centers and heel-to-toe time over 5 consecutive footsteps	MLP	10	96.2
Suutala, 2005, [53]	ElectroMechanical Film, floor	Features from spatial, frequency domain over a footsteps, combine different feature presentations for a footstep, and then combine on multiple footsteps	MLP, 1 footstep MLP, 5 footsteps	11	79.2 95.0
Suutala, 2008, [30]	Switch sensors, floor	Spatial, statistical, time-related features over a footsteps, stride length and cadence from multiple consecutive footsteps	GP, 1 footstep GP, 5–7 footsteps	9	64.2 84.3
Suutala, 2008, [44]	ElectroMechanical Film, floor	Features from spatial, frequency domain over a footsteps, combine different feature presentations for a footstep, and then combine on multiple footsteps	MLP, 1 footstep SVM, 2 footsteps SVM, 5 footsteps	10	63.3 81.9 91.7
Yun, 2008, [37]	Photo interrupters, floor	Array of sampled transitional footprints over 4 consecutive footsteps	MLP	10	92.0
Qian, 2008, [23]	Force Sensing Resistor, floor	1D pressure profile + 2D COP trajectory over a footstep, stride length, cadence, mean pressure of both footsteps	FLD	10	94.2
Qian, 2010, [24]	Force Sensing Resistor, floor	1D pressure profile + 2D COP trajectory over a footstep, stride length, mean pressure of both footsteps	FLD	11	92.3
The Proposed method	Photo interrupters, floor	Foot centers, heel-to-toe time, array of sampled transitional footprints over 5 consecutive footsteps, combine classifiers	MLP	10	99.0
